# Direct comparison of predictive performance of PRECISE-DAPT versus PARIS versus CREDO-Kyoto: a subanalysis of the ReCre8 trial

**DOI:** 10.1007/s12471-020-01486-y

**Published:** 2020-09-21

**Authors:** R. Rozemeijer, W. P. van Bezouwen, N. D. van Hemert, J. A. Damen, S. Koudstaal, M. Stein, G. E. Leenders, L. Timmers, A. O. Kraaijeveld, K. Roes, P. Agostoni, P. A. Doevendans, P. R. Stella, M. Voskuil

**Affiliations:** 1 Department of Cardiology, University Medical Center Utrecht, Utrecht, The Netherlands; 2 Julius Center for Health Sciences and Primary Care, University Medical Center Utrecht, Utrecht, The Netherlands; 3 Farr Institute of Health Informatics, University College London, London, UK; 4 Department of Cardiology, Zuyderland Medical Center, Heerlen, The Netherlands; 5 St. Antonius Hospital, Nieuwegein, The Netherlands; 6 Department of Biostatistics and Research Support, University Medical Center Utrecht, Utrecht, The Netherlands; 7 Department of Cardiology, Hartcentrum, Ziekenhuis Netwerk Antwerpen Middelheim, Antwerp, Belgium; 8 Netherlands Heart Institute, Utrecht, The Netherlands; 9 Central Military Hospital, Utrecht, The Netherlands

**Keywords:** Coronary artery disease, Risk stratification, Bleeding, Thrombosis, Percutaneous coronary intervention

## Abstract

**Background:**

Multiple scores have been proposed to guide risk stratification after percutaneous coronary intervention. This study assessed the performance of the PRECISE-DAPT, PARIS and CREDO-Kyoto risk scores to predict post-discharge ischaemic or bleeding events.

**Methods:**

A total of 1491 patients treated with latest-generation drug-eluting stent implantation were evaluated. Risk scores for post-discharge ischaemic or bleeding events were calculated and directly compared. Prognostic performance of both risk scores was assessed with calibration, Harrell’s c‑statistics net reclassification index and decision curve analyses.

**Results:**

Post-discharge ischaemic events occurred in 56 patients (3.8%) and post-discharge bleeding events in 34 patients (2.3%) within the first year after the invasive procedure. C‑statistics for the PARIS ischaemic risk score was marginal (0.59, 95% confidence interval (CI) 0.51–0.68), whereas the CREDO-Kyoto ischaemic risk score was moderate (0.68, 95% CI 0.60–0.75). With regard to post-discharge bleeding events, CREDO-Kyoto displayed moderate discrimination (c-statistic 0.67, 95% CI 0.56–0.77), whereas PRECISE-DAPT (0.59, 95% CI 0.48–0.69) and PARIS (0.55, 95% CI 0.44–0.65) had a marginal discriminative capacity. Net reclassification index and decision curve analysis favoured CREDO-Kyoto-derived bleeding risk assessment.

**Conclusion:**

In this contemporary all-comer population, PARIS and PRECISE-DAPT risk scores were not resilient to independent testing for post-discharge bleeding events. CREDO-Kyoto-derived risk stratification was associated with a moderate predictive capability for post-discharge ischaemic or bleeding events. Future studies are warranted to improve risk stratification with more focus on robustness and rigorous testing.

**Electronic supplementary material:**

The online version of this article (10.1007/s12471-020-01486-y) contains supplementary material, which is available to authorized users.

## What’s new?

- Currently available risk scores may improve stratification for the risk of post-discharge events. Performance data and analyses with a direct comparison of risk scores are, however, limited.
- We used a contemporary cohort of patients to calculate three risk scores to compare net reclassification indices, receiver operating characteristic curves and decision curves.
- CREDO-Kyoto-derived risk stratification had a moderate predictive performance for post-discharge ischaemic or bleeding events.
- PARIS-derived and PRECISE-DAPT-derived risk stratifications were not resilient to independent testing for the risk of post-discharge events.
- Future studies are warranted to improve risk stratification with focus on risk score robustness and rigorous testing in external datasets.

## Introduction

Dual antiplatelet therapy (DAPT), consisting of aspirin and a P2Y_12_ inhibitor, represents the cornerstone of treatment for patients with acute coronary syndrome or after percutaneous coronary intervention (PCI) with drug-eluting stent implantation [1–3]. DAPT mitigates the risk of ischaemic events [4], but this is counterbalanced by an increased risk of bleeding events [5], mainly gastro-intestinal bleeding.

The risk scores of PRECISE-DAPT [6], PARIS [7] and CREDO-Kyoto [8] have been developed specifically to assess the risks of both post-discharge ischaemic and bleeding events following PCI. Although all three risk scores were moderately accurate in their derivation cohorts (c-statistics of ~0.65 to 0.70), they remain poorly characterised in external cohorts. Indeed, the prognostic performance has not yet been directly compared. Accordingly, we aimed to assess and to directly compare the predictive performance of currently used risk scores for post-discharge ischaemic or bleeding events in a contemporary all-comer population.

## Methods

### Study design and patient population

The present study is a subanalysis of the physician-initiated, prospective, multicentre Randomised All-Comers Evaluation of a Permanent Polymer Zotarolimus-Eluting Stent Versus a Polymer-Free Amphilimus-Eluting Stent (ReCre8) trial, as previously reported [9, 10]. In brief, the ReCre8 trial was designed to evaluate clinical non-inferiority of the polymer-free amphilimus-eluting stent as compared with a latest-generation permanent polymer zotarolimus-eluting stent in a 1:1 ratio across three European centres. Between 3 November, 2014 and 10 July, 2017, consecutive patients were randomly allocated to a stent group after stratification for troponin status and presence of diabetes mellitus. In both treatment arms, troponin-positive patients were planned for 12-month DAPT, whereas troponin-negative patients were planned for 1‑month DAPT. Inclusion criteria were broad, while exclusion criteria were minor to reflect routine clinical practice.

The protocol was approved by the Medical Research Ethics Committee Utrecht and the institutional review board of each participating centre and monitored by an independent clinical research organisation (Julius Clinical Research, Zeist, the Netherlands). Clinical endpoints were defined according to the Academic Research Consortium criteria [11] and Bleeding Academic Research Consortium criteria [12] and adjudicated by an independent clinical event committee, with complete verification of source documents. Post-discharge events were defined as adverse events occurring 2 or more days after the index procedure. This study complied with the principles of the Declaration of Helsinki and was reported according to the Transparent Reporting of a multivariable prediction model for Individual Prognosis Or Diagnosis (TRIPOD) statement [13]. Written informed consent was obtained from each patient that participated in this study.

### Risk scores

**Fig. 1:**
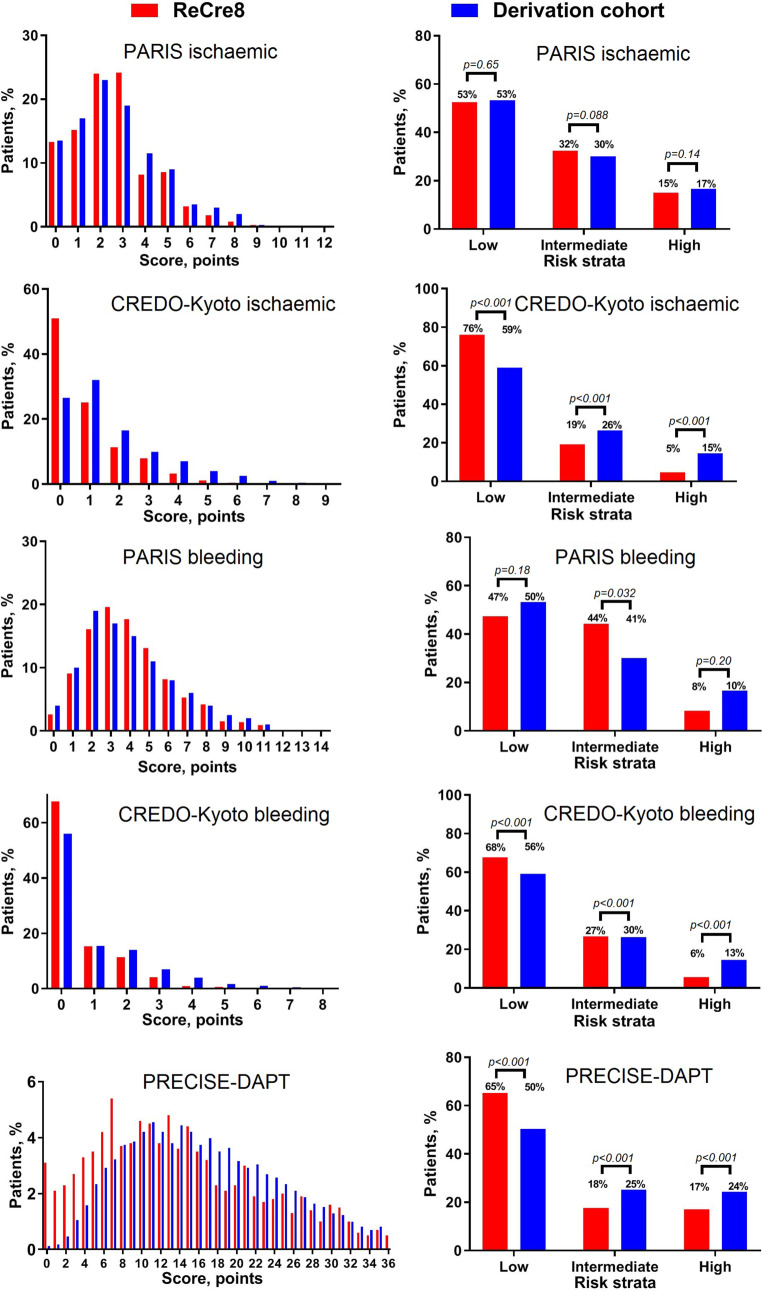
Distribution of contemporary risk scores. Risk strata were evaluated using χ^2^ test, with established definitions [6–8]

The PRECISE-DAPT [6], PARIS [7] and CREDO-Kyoto risk scores [8] were calculated and assigned to each patient using established definitions (see files in the Electronic Supplementary Material: Tab. 1 and 2 and Fig. 1). To enable comparisons between the PRECISE-DAPT and the other risk scores, we categorised patients into three risk strata (i.e. low, intermediate and high risk) by considering ‘very low risk’ and ‘low risk’ as one risk stratum. Creatinine clearance was calculated using the Cockcroft-Gault formula [14]. Anaemia was defined as a haemoglobin level <7.0 mmol/L for women and <7.5 mmol/L for men [15].

### Statistical analysis

Continuous variables are expressed as mean ± standard deviation (SD), and binary variables as counts (*n*) and percentages (%). Differences were tested using the Wilcoxon rank-sum test and the Student’s *t*-test for continuous variables, or the χ^2^ test as appropriate. First, risk score distributions were visualised graphically. Patients were categorised into different risk strata (i.e. low, intermediate and high risk). The ability to discriminate between low- and high-risk strata for both risk scores regarding post-discharge ischaemic or bleeding events was evaluated using Kaplan-Meier estimates of time to first post-discharge event with log-rank tests [16].

Indices of calibration and discrimination were used to assess predictive performance. Calibration (i.e. the degree to which the estimated risks match the observed risks) was assessed and plotted as observed versus predicted outcomes for each risk stratum of each risk score. To assess model discrimination (i.e. the ability to distinguish patients at low, intermediate or high risk), receiver operating characteristic curves and Harrell’s c‑statistics with 95% confidence interval (CI) were calculated and compared using the non-parametric approach of DeLong [17]. The net reclassification improvement index was calculated and decision curve analysis [18] was used to determine a possible net benefit (i.e. the balance between the number of true positives and false positives) over a range of cut-off values: the higher the net benefit, the better the risk score. Theoretically, cut-off values should range from negative infinity to the incidence of the disease or outcome of interest.

A complete-case analysis was performed since the number of missing values was low (<2.5%). *P*-values were two-sided and were considered statistically significant when *p* < 0.05. Statistical analyses were performed using SAS version 9.4 (SAS Institute, Cary, USA) and R version 3.4.1 (R Foundation for Statistical Computing, Vienna, Austria). Figures were generated using GraphPad Prism version 7.04 (GraphPad Inc., San Diego, CA, USA) and R version 3.4.1.

## Results

### Baseline characteristics and risk score distribution

The ReCre8 trial population comprised 1491 patients and was characterised by a lower number of diabetic patients (20% vs 34%, 41% and 28%) and a higher number of troponin-positive acute coronary syndrome patients (40% vs 8%, 15% and 32%) than the derivation cohorts of the PARIS, CREDO-Kyoto and PRECISE-DAPT risk scores (Tab. 1). Compared with the PARIS cohort, current smoking was more prevalent (25.8% vs 17.8%), but anaemia was less prevalent (6.5% vs 15.5%). Fewer patients had renal insufficiency (11.0% vs 15.8%). Compared with the CREDO-Kyoto cohort, more patients in the ReCre8 trial had a history of myocardial infarction (19.9% vs 13.4%). The baseline characteristics of the PRECISE-DAPT cohort were generally similar, although the ReCre8 trial population had a lower incidence of prior bleeding (1.3% vs 1.9%).

**Table 1:** Baseline characteristics of ReCre8 trial population, compared with derivation cohorts for PARIS, CREDO-Kyoto and PRECISE-DAPT risk scores

Characteristic	ReCre8 (*n* = 1491)	PARIS (*n* = 4190)	*p*-value^a^	CREDO-Kyoto (*n* = 4778)	*p*-value^b^	PRECISE-DAPT (*n* = 14,963)	*p*-value^c^
*Clinical characteristics*							
Age, years	64.9 ± 11.0	63.6 ± 11.0	0.50	68.1 ± 10.3	<0.001	65.0 (56.9–73.0)	–
Female	349 (23.4)	1072 (25.4)	0.088	1331 (27.8)	<0.001	4414 (29.5)	<0.001
Body mass index, kg/m^2^	27.3 ± 4.4	29.3 ± 5.5	<0.001	23.8 ± 3.4	<0.001	N/A	–
Current smoker	384 (25.8)	745 (17.8)	<0.001	1322 (27.6)	0.15	3757 (28.0)	–
Diabetes mellitus	304 (20.4)	1422 (34.1)	<0.001	1952 (40.9)	<0.001	4168 (27.9)	<0.001
Insulin-treated	96 (6.4)	473 (11.2)	<0.001	499 (10.4)	<0.001	797 (5.4)	0.24
Low eGFR (<60 ml/min per 1.73 m^2^)^d^	164 (11.0)	663 (15.8)	<0.001	374 (7.9)^e^	<0.001	N/A	–
WBC count (10^3^ units/μL)	8.2 (6.6–10.6)	N/A	–	N/A	–	7.8 (6.3–10.2)	–
Anaemia^f^	97 (6.5)	653 (15.5)	<0.001	517 (10.8)	<0.001	N/A	–
Low platelet count (<100 × 10^9^/L)	11 (0.7)	N/A	–	63 (1.3)	0.089	N/A	–
Triple therapy	120 (8.0)	202 (4.8)	<0.001	389 (8.1)	0.90	N/A	–
Impaired LVEF	278 (18.6)	N/A	–	772 (16.2)	0.06	N/A	–
Peripheral vascular disease	N/A	334 (8.0)	–	371 (7.8)	–	714 (10.4)	–
Malignancy	N/A	N/A	–	408 (8.5)	–	N/A	–
*Relevant medical history*							
Prior MI	297 (19.9)	1044 (24.9)	<0.001	641 (13.4)	<0.001	2946 (19.8)	0.86
Prior PCI	304 (20.4)	1758 (41.9)	<0.001	0 (0)	–	2392 (16.0)	0.54
Prior CABG	138 (9.3)	603 (14.3)	<0.001	0 (0)	–	893 (6.0)	<0.001
Prior bleeding^g^	19 (1.3)	N/A	–	N/A	–	82 (1.9)	0.001
*Clinical presentation*							
Stable angina	633 (42.2)	2622 (62.5)	<0.001	N/A	–	6299 (44.4)	0.98
Unstable angina	109 (7.2)	1254 (29.9)	<0.001	N/A	–	3215 (22.7)	<0.001
NSTEMI or STEMI	599 (40.2)	331 (7.8)	<0.001	733 (15.3)	<0.001	4669 (32.2)	<0.001
*Procedural features*							
Chronic total occlusion	98 (6.6)	N/A	–	633 (13.2)	<0.001	N/A	–

Data are *n* (%), mean ± standard deviation, or median (interquartile range)

*CABG* coronary artery bypass grafting, *eGFR* estimated glomerular filtration rate, *LVEF* left ventricular ejection fraction, *MI* myocardial infarction, *N/A* not available, *NSTEMI* non-ST-segment elevation myocardial infarction, *PCI* percutaneous coronary intervention, *STEMI* ST-segment elevation myocardial infarction, *WBC* white blood cell

^a^*P*-value for comparison of ReCre8 and PARIS cohorts

^b^*P*-value for comparison of ReCre8 and CREDO-Kyoto cohorts

^c^*P*-value for comparison of ReCre8 and PRECISE-DAPT cohorts

^d^eGFR was calculated using the Cockcroft-Gault formula

^e^Defined as eGFR <30 ml/min per 1.73 m^2^

^f^Defined as haemoglobin levels <7.0 mmol/L for women and <7.5 mmol/L for men

^g^Defined as history of previous clinically significant bleeding requiring medical attention

The risk score distributions in our cohort were generally comparable to those of the derivation cohorts of the PARIS, CREDO-Kyoto and PRECISE-DAPT risk scores (Fig. 1). The proportion of patients that were classified into the low, intermediate and high ischaemic risk strata using the PARIS ischaemic risk score in the ReCre8 trial population were similar to the PARIS derivation cohort. Conversely, using PRECISE-DAPT to stratify our trial population, less patients were categorised as low risk (65% vs 50%) or high risk (17% vs 24%) compared with the PRECISE-DAPT derivation cohort.

### Post-discharge adverse events

**Fig. 2:**
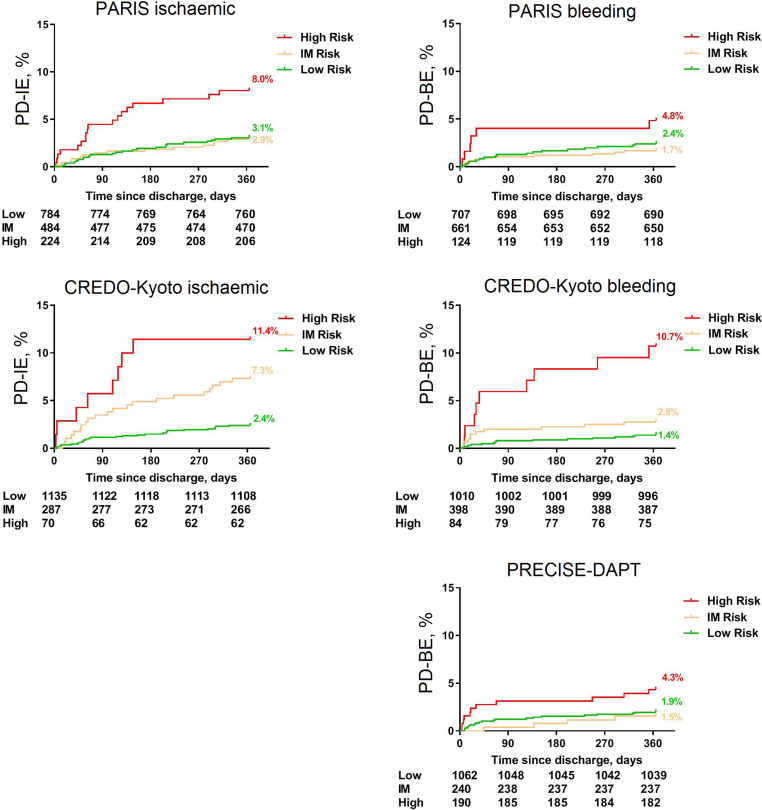
One-year Kaplan-Meier curves for various risk strata using contemporary risk scores. *IM* intermediate, *PD-BE* post-discharge bleeding events, *PD-IE* post-discharge ischaemic events

One-year Kaplan-Meier curves assessing the divergence of post-discharge ischaemic events over the risk strata showed significant differences for PARIS (log-rank *p* < 0.001), CREDO-Kyoto (log-rank *p* < 0.001) and PRECISE-DAPT (log-rank *p* < 0.001) (Fig. 2). However, some overlap was seen in the low- and intermediate-risk stratum using the PARIS ischaemic risk score and the PRECISE-DAPT risk score. In the present study post-discharge ischaemic events occurred in 56 patients (3.8%) and post-discharge bleeding events in 34 patients (2.3%).

Cardiac death occurred in 18 patients (1.2%), 14 patients suffered a myocardial infarction (0.9%), 6 had a definite or probable stent thrombosis (0.4%) and 8 had an ischaemic stroke (0.5%). Post-discharge bleeding events occurred in 34 patients (2.3%) within the first year after the index PCI.

### Predictive performance of ischaemic risk scores

**Fig. 3:**
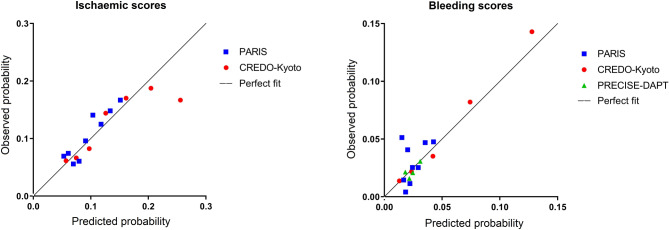
Expected and observed rates of post-discharge events using contemporary risk scores. A perfect fit (diagonal line) represents predicted probability being equal to observed probability. Points under this line (predicted probability > observed probability) indicate an overestimation of the risk of post-discharge events, while points above this line (predicted probability > observed probability) indicate an underestimation of the risk of post-discharge events

**Fig. 4:**
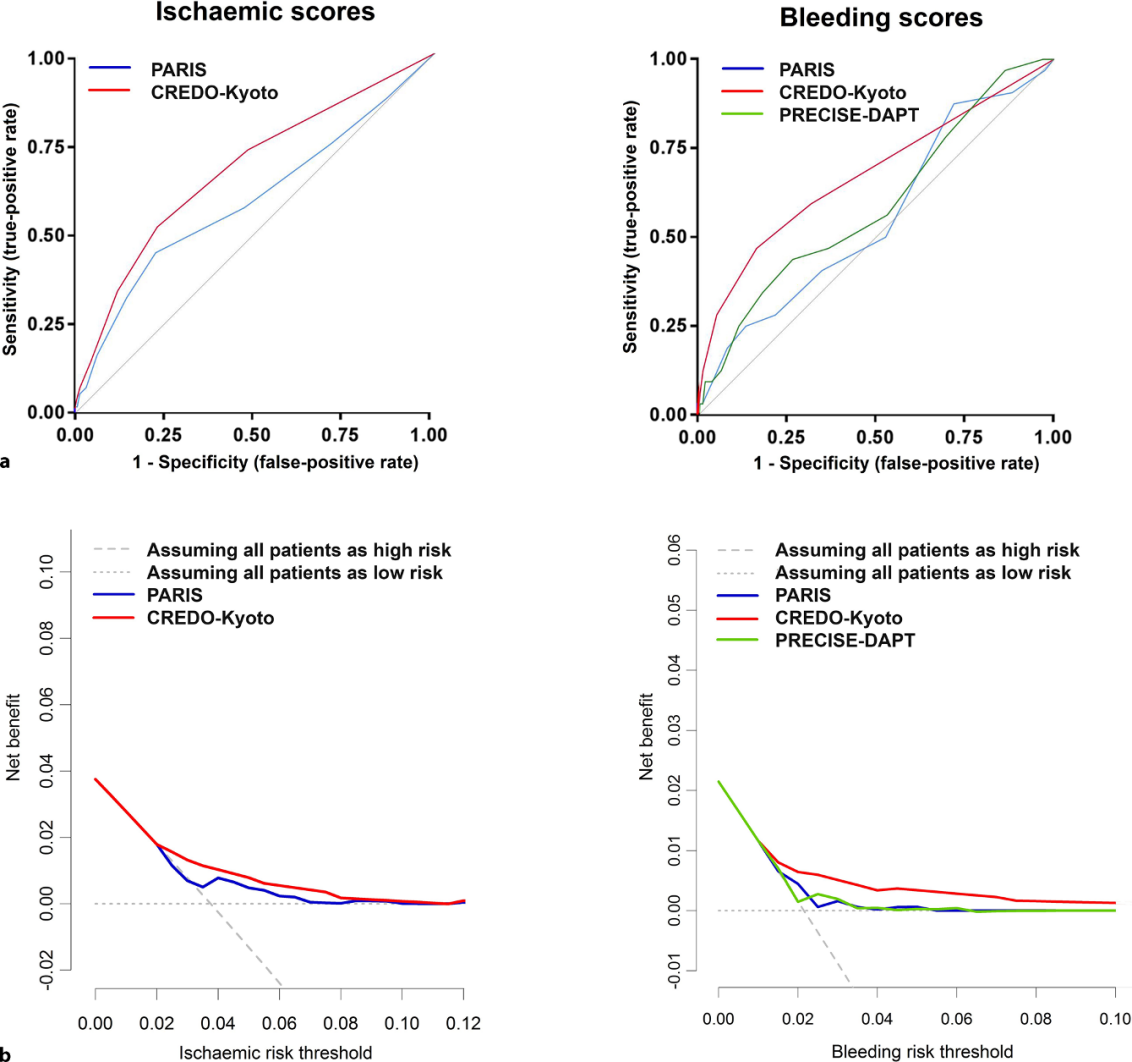
Receiver operator characteristic curves (**a**) and decision curves (**b**) of contemporary risk scores. **a** Diagonal line indicates random classification (probability of true-positive rate being similar to probability of false-positive rate). Values above the diagonal line indicate better prediction of true positives. A larger area under the curve indicates a better discriminative performance of the risk prediction model. **b** Net benefit indicates difference between true-positive and false-positive rate, corrected for threshold probability (x-axis). Higher values indicate better predictive performance of the model. Grey lines represent stratification when no model was used

Calibration of the ischaemic risk scores appeared reasonable for both PARIS and CREDO-Kyoto with regard to the predicted post-discharge ischaemic event rates (Fig. 3). Discriminative power and reclassification are summarised in Tab. 2. Discriminative performance of PARIS (Fig. 4a) was marginal (c-statistic 0.59, 95% CI 0.51–0.68), whereas that of CREDO-Kyoto was moderate (0.68, 95% CI 0.60–0.75). Reclassification with CREDO-Kyoto resulted in a lower number of cases in the high-risk stratum (i.e. fewer patients with post-discharge ischaemic events were classified in high-risk categories by CREDO-Kyoto) than with PARIS (see Tab. 4 in the Electronic Supplementary Material).

**Table 2:** Discriminative power of risk scores for post-discharge adverse events

**Post-discharge ischaemic risk scores**	**PARIS**	**CREDO-Kyoto**	
Hazard ratio^a^	1.15 (95% CI 1.05 to 1.27)	1.33 (95% CI 1.17 to 1.52)	
Harrell’s c‑statistic	0.59 (95% CI 0.51 to 0.68)	0.68 (95% CI 0.60 to 0.75)	
Comparing c‑statistic	Ref	*p* = 0.47^b^	
NRI (cases)	Ref	−0.17 (−0.32 to −0.19)^c^	
NRI (non-cases)	Ref	0.28 (0.25 to 0.31)^c^	
**Post-discharge bleeding risk scores**	**PARIS**	**CREDO-Kyoto**	**PRECISE-DAPT**
Hazard ratio^a^	1.10 (95% CI 0.96 to 1.26)	1.82 (95% CI 1.44 to 2.31)	1.02 (0.99 to 1.05)
Harrell’s c‑statistic	0.55 (95% CI 0.44 to 0.65)	0.67 (95% CI 0.56 to 0.77)	0.59 (0.48 to 0.69)
Comparing c‑statistic	Ref	*p* = 0.075^b^	*p* = 0.46^b^
NRI (cases)	Ref	0.13 (−0.09 to 0.34)^c^	0.06 (−0.13 to 0.25)^c^
NRI (non-cases)	Ref	0.21 (0.18 to 0.24)^c^	0.11 (0.08 to 0.14)^c^

*NRI* net reclassification improvement index, *95% CI* 95% confidence interval

^a^Using risk scores (continuous variables) as a global prognostic indicator by Cox models

^b^Comparing c‑statistic with PARIS as reference

^c^Comparing NRI with PARIS as reference

CREDO-Kyoto was able to enhance risk stratification on post-discharge ischaemic events in all-comer patients treated with latest-generation drug-eluting stent implantation as compared with the situation in which no model was used (Fig. 4b). When compared with the situation of assuming all patients as low risk, CREDO-Kyoto enhanced stratification at a risk threshold of ≥2.0%. Applying CREDO-Kyoto when using a risk threshold of 4.0% resulted in a net benefit of +1.0%, while assuming all patients as high risk resulted in a net benefit of −0.3%; for PARIS, this resulted in a net benefit of +0.5% (see Tab. 5 in the Electronic Supplementary Material). In other words, the net benefit using CREDO-Kyoto-derived risk stratification for post-discharge ischaemic events at a risk threshold of 4.0% led to the equivalent of 30.8 (PARIS 24.8) and 24.7 (PARIS 18.0) more true positives per 100 patients than the assumption of all patients being high risk or low risk, respectively.

### Predictive performance of bleeding risk scores

Calibration of the observed probability approximated the predicted probability more closely for CREDO-Kyoto and PRECISE-DAPT than for PARIS (Fig. 3), which revealed some degree of underestimation and overestimation of post-discharge bleeding event rates. Discriminative capacity and reclassification are summarised in Tab. 2. PARIS-derived (c-statistic 0.55, 95% CI 0.44–0.65) and PRECISE-DAPT-derived risk stratification (c-statistic 0.59; 95% CI 0.48–0.69) (Fig. 4a) did not enhance risk stratification to distinguish patients with a low risk of post-discharge bleeding events from those with a high risk. CREDO-Kyoto had a moderate discriminative capacity (c-statistic 0.67, 95% CI 0.56–0.77) to predict post-discharge bleeding events. Using PARIS as reference, the net reclassification improvement index revealed a more accurate classification with CREDO-Kyoto, but not with PRECISE-DAPT (see Tab. 4 in the Electronic Supplementary Material).

Using CREDO-Kyoto-derived risk stratification, we were able to more accurately predict post-discharge bleeding events in all-comer patients with latest-generation drug-eluting stents, compared with the situation in which PARIS, PRECISE-DAPT or no model was used (Fig. 4b). Based on decision curve analysis, CREDO-Kyoto-derived risk stratification performed better than PARIS or PRECISE-DAPT. Especially when a risk threshold of ≥2% was chosen, the net benefit of CREDO-Kyoto outperformed PARIS, PRECISE-DAPT or the assumption of all patients being high risk or low risk (see Tab. 5 in the Electronic Supplementary Material). In other words, the net benefit using CREDO-Kyoto at a risk threshold of 4%, for example, led to the equivalent of 54.4 (PARIS 46.8 vs PRECISE-DAPT 47.5) and 8.1 more true positives per 100 patients (PARIS 0.5 vs PRECISE-DAPT 1.1) than assuming all patients as high risk or low risk, respectively.

## Discussion

In this study, we assessed and directly compared the predictive performance of the PRECISE-DAPT, PARIS and CREDO-Kyoto risk scores  for post-discharge events in a contemporary cohort of patients following latest-generation drug-eluting stent implantation. The main findings were: (1) CREDO-Kyoto-derived risk stratification was associated with a moderate predictive performance with respect to post-discharge events; and (2) PARIS-derived and PRECISE-DAPT-derived risk stratifications had a marginal discriminative capacity to adequately define the risk of post-discharge events in unselected patients.

The discriminative capability of CREDO-Kyoto was roughly similar to that of the validation cohort (c-statistic for ischaemic events 0.68 vs 0.64; c‑statistic for bleeding events 0.67 vs 0.66) [8]. Based on our data, CREDO-Kyoto may especially aid clinicians who need support in their clinical judgment regarding patients with a low to intermediate risk of post-discharge events. The absence of peripheral artery disease and malignancy may have introduced a slight underestimation in terms of a 1- to 2‑point left-shift in the CREDO-Kyoto risk score, in approximately 10 to 15% of the patients [19]. Remarkably, this did not heavily impact the predictive capability of CREDO-Kyoto in the present analysis. The finding of a robust c‑statistic while using less parameters, underscores the relevance of a parsimonious approach when developing a risk prediction model to optimise clinical utility.

The lower-than-expected prognostic performance of PRECISE-DAPT and PARIS for post-discharge events is largely consistent with a previous report that found a c-statistic of 0.61 for PARIS and of 0.63 for PRECISE-DAPT [20]. Noteworthy, this cohort was comprised of patients with acute coronary syndrome, whereas our cohort included roughly one-half of patients with stable coronary artery disease and one-half of patients with acute coronary syndrome. Several issues may have contributed to the limited prognostic capability and should be borne in mind when interpreting our results. For instance, procedural parameters (i.e. chronic total occlusions, bifurcations, implantation of more than 3 stents or treatment of more than 3 lesions) were neglected in these risk scores, but are in fact related to adverse events [21, 22].

Another important limitation of currently available risk scores is that stent specific parameters are not accounted for. For example, the PRECISE-DAPT risk score was derived from a cohort in which patients were treated with 13% bare-metal stents, whereas the ReCre8 trial was comprised of a cohort that was solely treated with contemporary latest-generation drug-eluting stents which may have a lower ischaemic risk profile [10].

The moderate performance of risk scores in the present study may also be explained by the composition and incidence of the incorporated risk factors [23], as related to the patient’s true baseline risks. For example, none of the contemporary risk scores address the importance of lesion complexity, which also affects the risk of post-discharge events [24]. Reconciliation of such differences remains challenging and largely reflects the variety in PCI populations.

The bleeding criteria of the Bleeding Academic Research Consortium (BARC) were used to define bleeding in the present study and in the PARIS registry, as opposed to the PRECISE-DAPT study, where bleeding definitions were based on criteria for thrombolysis in myocardial infarction. This complicated the present comparison and may have underestimated the bleeding risk. The BARC criteria are, however, currently considered the standard bleeding definition [25].

Finally, rigorous testing of contemporary risk scores is essential for risk stratification, since the generalisability of risk scores remains an important drawback and is often poorly characterised. It should not be forgotten that circa 350 risk models have been investigated in cardiovascular disease over the last decades [26], but that not more than a handful were resilient to independent testing [27–29]. Future studies should aim to elucidate the role of contemporary risk scores in a prospective randomised setting to evaluate their impact on clinical outcomes.

### Study limitations

This study has several limitations. First, it should be noted that we addressed risk factors at the time of the index PCI. However, it seems more likely that a patient’s risk is not static, but may vary over time [30]. Second, we obtained data over a 1-year follow-up period, whereas the risk scores were derived from data with a follow-up time of up to 3 years. This may have slightly underestimated the predictive performance of the risk scores in our cohort, although the first year after PCI is the period of greatest ischaemic and haemorrhagic risk. Third, risk stratification may have been underestimated regarding post-discharge ischaemic events and overestimated in terms of post-discharge bleeding events in troponin-negative patients because of a different regimen of DAPT. However, various DAPT strategies (e.g. 3 to 12 months) were present in the cohorts used to derive the three risk scores.

## Conclusion

Based on this all-comer population treated with latest-generation drug-eluting stents, the PRECISE-DAPT and PARIS risk scores were not resilient to independent testing for patients’ risks of post-discharge bleeding events. CREDO-Kyoto-derived risk stratification was associated with a moderate predictive capability regarding post-discharge ischaemic or bleeding events. Future studies are warranted to improve risk assessment and to prospectively evaluate the impact on clinical outcomes when used to individualise DAPT.

## Caption Electronic Supplementary Material

Fig. 1 PRECISE-DAPT score^3^

Tab. 1 Risk scores from PARIS^1^

Tab. 2 CREDO-Kyoto risk scores^2^

Tab. 3 Outcome definitions used in contemporary risk scores

Tab. 4 Net Reclassification tables of contemporary risk scores

Tab. 5 Net benefit tables for contemporary risk scores
