# Supplementary material for: Direct comparison of predictive performance of PRECISE-DAPT versus PARIS versus CREDO-Kyoto: a subanalysis of the ReCre8 trial
Source: Neth Heart J. 2020 Sep 21;29(4):201–14. doi: 10.1007/s12471-020-01486-y (PMC7991032; doi:10.1007/s12471-020-01486-y)
Supplement: Supplementary file 1 — Fig. 1 PRECISE-DAPT score3 [file 12471_2020_1486_MOESM1_ESM.docx]

**Electronic Supplementary Material**

**Fig. 1** PRECISE-DAPT score^3^

**
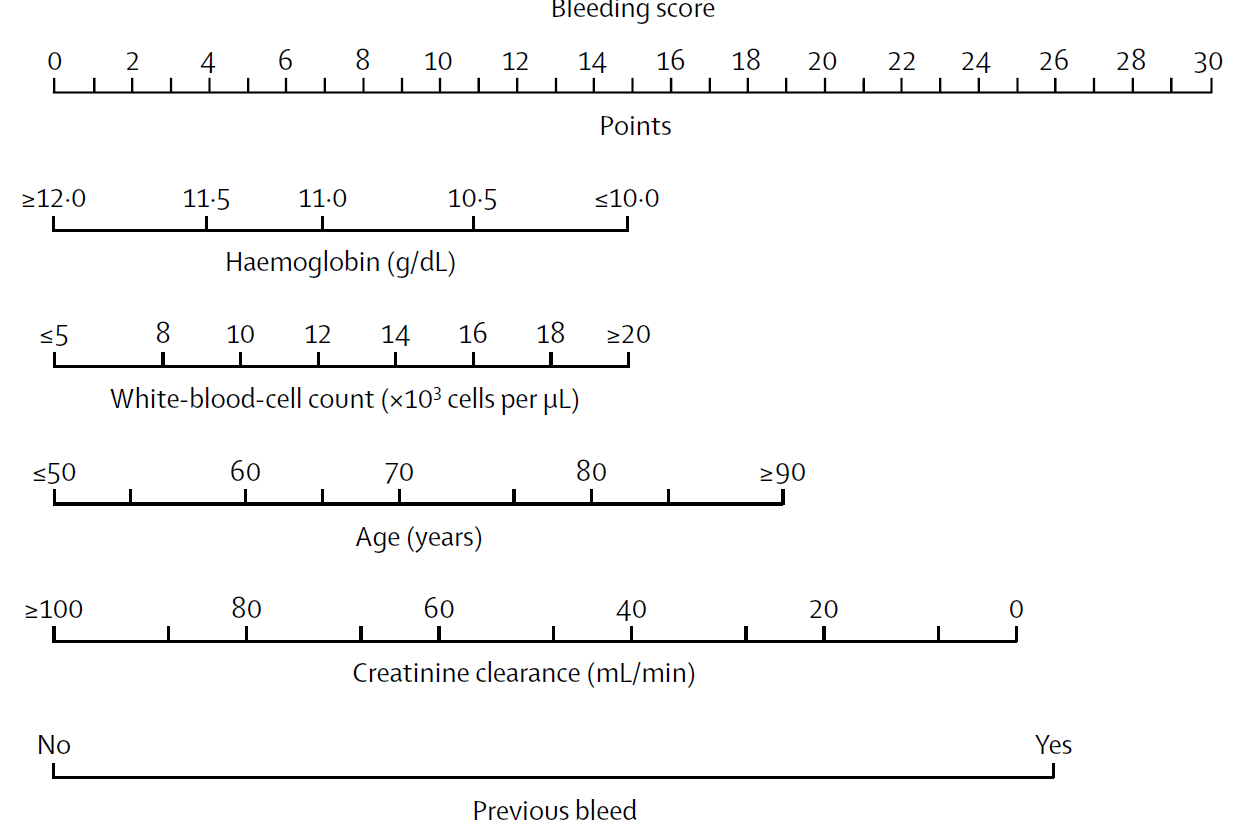
**

**Reference**

3. Costa F, van Klaveren D, James S, Heg D, Raber L, Feres F, Pilgrim T, Hong MK, Kim HS, Colombo A, Steg PG, Zanchin T, Palmerini T, Wallentin L, Bhatt DL, Stone GW, Windecker S, Steyerberg EW, Valgimigli M and Investigators P-DS. Derivation and validation of the predicting bleeding complications in patients undergoing stent implantation and subsequent dual antiplatelet therapy (PRECISE-DAPT) score: a pooled analysis of individual-patient datasets from clinical trials. *Lancet*. 2017;389:1025-1034.
