# Supplementary material for: Direct comparison of predictive performance of PRECISE-DAPT versus PARIS versus CREDO-Kyoto: a subanalysis of the ReCre8 trial
Source: Neth Heart J. 2020 Sep 21;29(4):201–14. doi: 10.1007/s12471-020-01486-y (PMC7991032; doi:10.1007/s12471-020-01486-y)
Supplement: Supplementary file 2 — Tab. 1 Risk scores from PARIS1 [file 12471_2020_1486_MOESM2_ESM.docx]

**Electronic Supplementary Material**

**Tab. 1** Risk scores from PARIS^1^

| PARIS ischemic risk score |  |  | PARIS bleeding risk score |  |  |
| --- | --- | --- | --- | --- | --- |
| Parameter | **Score** |  | **Parameter** | **Score** |  |
| Diabetes Mellitus |  |  | Age |  |  |
| none | 0 |  | <50 | 0 |  |
| non-insulin dependent | +1 |  | 50-59 | +1 |  |
| insulin-dependent | +3 |  | 60-69 | +2 |  |
| Acute Coronary Syndrome |  |  | 70-79 | +3 |  |
| no | 0 |  | ≥80 | +4 |  |
| yes, troponin negative | +1 |  | BMI, kg/m^2^ |  |  |
| yes, troponin positive | +2 |  | <25 | +2 |  |
| Current Smoking |  |  | 25-34.9 | 0 |  |
| no | 0 |  | ≥35 | +2 |  |
| yes | +1 |  | Current Smoking |  |  |
| CrCl <60ml/min |  |  | no | 0 |  |
| absent | 0 |  | yes | +2 |  |
| present | +2 |  | Anemia |  |  |
| Prior PCI |  |  | absent | 0 |  |
| no | 0 |  | present | +3 |  |
| yes | +2 |  | CrCl <60ml/min |  |  |
| Prior CABG |  |  | absent | 0 |  |
| no | 0 |  | present | +2 |  |
| yes | +2 |  | Triple therapy on discharge |  |  |
|  |  |  | no | 0 |  |
|  |  |  | yes | +2 |  |
| total score range: 0-12 |  |  | **total score range: 0-15** |  |  |

BMI = body mass index, CrCl = Creatinine Clearance, CABG = coronary artery bypass grafting, PCI = Percutaneous Coronary Intervention.

**PARIS ischemic risk strata**

The ischemic risk score from PARIS (range: 0 to 12) was divided into a low-risk (ischemic score 0-2), intermediate-risk (ischemic score 3-4) and high-risk group (ischemic score ≥5) for post-discharge ischemic events.

**PARIS bleeding risk strata**

The PARIS bleeding score ranges from 0 to 15, with three risk strata: low-risk (score 0-3), intermediate-risk (score 4-7), and high-risk (score ≥8) for post-discharge major bleeding events.

**Reference**

1. Baber U, Mehran R, Giustino G, Cohen DJ, Henry TD, Sartori S, Ariti C, Litherland C, Dangas G, Gibson CM, Krucoff MW, Moliterno DJ, Kirtane AJ, Stone GW, Colombo A, Chieffo A, Kini AS, Witzenbichler B, Weisz G, Steg PG and Pocock S. Coronary Thrombosis and Major Bleeding After PCI With Drug-Eluting Stents: Risk Scores From PARIS. *J Am Coll Cardiol*. 2016;67:2224-2234.
