# Supplementary material for: Direct comparison of predictive performance of PRECISE-DAPT versus PARIS versus CREDO-Kyoto: a subanalysis of the ReCre8 trial
Source: Neth Heart J. 2020 Sep 21;29(4):201–14. doi: 10.1007/s12471-020-01486-y (PMC7991032; doi:10.1007/s12471-020-01486-y)
Supplement: Supplementary file 3 — Tab. 2 CREDO-Kyoto risk scores2 [file 12471_2020_1486_MOESM3_ESM.docx]

**Electronic Supplementary Material**

**Tab. 2** CREDO-Kyoto risk scores^2^

| CREDO-Kyoto ischemic risk score |  |  | CREDO-Kyoto bleeding risk score |  |  |
| --- | --- | --- | --- | --- | --- |
| Parameter | **Score** |  | **Parameter** | **Score** |  |
| Severe Chronic Kidney Disease | 2 |  | Platelet Count < 100 *10^9^/L | 2 |  |
| Atrial Fibrillation | 2 |  | Severe Chronic Kidney Disease | 2 |  |
| Peripheral Vascular Disease | 2 |  | Peripheral Vascular Disease | 2 |  |
| Anemia | 2 |  | Heart Failure | 2 |  |
| Age ≥ 75 years | 1 |  | Prior Myocardial Infarction | 1 |  |
| Heart Failure | 1 |  | Malignancy | 1 |  |
| Diabetes Mellitus | 1 |  | Atrial Fibrillation | 1 |  |
| Chronic Total Occlusion | 1 |  |  |  |  |
| total score range: 0-12 |  |  | **total score range: 0-11** |  |  |

**CREDO-Kyoto ischemic risk strata**

The CREDO-Kyoto ischemic score was designed to predict post-discharge ischemic events (range: 0 to 12), and categorizes patients in low-risk (ischemic score 0-1), intermediate-risk (ischemic score 2-3), and high-risk (ischemic score ≥4).

**CREDO-Kyoto bleeding risk strata**

The CREDO-Kyoto bleeding score (range: 0 to 11) aims to create risk strata (low: score 0; intermediate: score 1-2; high: score ≥3) for post-discharge bleeding events.

**Reference**

2. Natsuaki M, Morimoto T, Yamaji K, Watanabe H, Yoshikawa Y, Shiomi H, Nakagawa Y, Furukawa Y, Kadota K, Ando K, Akasaka T, Hanaoka KI, Kozuma K, Tanabe K, Morino Y, Muramatsu T, Kimura T, Credo-Kyoto Pci/Cabg Registry Cohort R and investigators Nt. Prediction of Thrombotic and Bleeding Events After Percutaneous Coronary Intervention: CREDO-Kyoto Thrombotic and Bleeding Risk Scores. *J Am Heart Assoc*. 2018;7.
