# Supplementary material for: Direct comparison of predictive performance of PRECISE-DAPT versus PARIS versus CREDO-Kyoto: a subanalysis of the ReCre8 trial
Source: Neth Heart J. 2020 Sep 21;29(4):201–14. doi: 10.1007/s12471-020-01486-y (PMC7991032; doi:10.1007/s12471-020-01486-y)
Supplement: Supplementary file 4 — Tab. 3 Outcome definitions used in contemporary risk scores [file 12471_2020_1486_MOESM4_ESM.docx]

**Electronic Supplementary Material**

**Tab. 3** Outcome definitions used in contemporary risk scores

| Outcomes | ReCre8^4^ | PARIS^1^ | CREDO-Kyoto^2^ | PRECISE-DAPT^3^ |
| --- | --- | --- | --- | --- |
| Myocardial Infarction | The typical rise and fall in cardiac markers of at least one time the URL. | The presence of clinical or ECG changes consistent with myocardial ischemia in the setting of increased cardiac biomarkers greater than the URL | The presence of clinical symptoms, ECG change or abnormal imaging findings of myocardial infarction combined with an increase in CK-MB greater than three URL or troponin-T/troponin-I more than the 99th percentile of URL. | Not used. |
| ST (definite or probable) | According to the ARC Criteria. | According to the ARC Criteria. | According to the ARC Criteria. | Not used. |
| Ischemic stroke | Stroke confirmed by a neurologist with symptoms persisted more than 24 hours. | Not used as outcome. | Stroke requiring hospitalization with symptoms lasting >24 hours. | Not used. |
| Bleeding | According to BARC criteria, category 2, 3 or 5 bleedings. | According to BARC criteria, category 3 or 5 bleedings. | According to the GUSTO criteria, moderate or severe bleedings. | According to TIMI criteria, major or minor bleedings. |
|  |  |  |  |  |
| Follow-up period | Post-discharge up to 12 months. | Post-discharge up to 24 months. | Post-discharge up to 36 months. | 7 days after index up to 12 months. |

ARC = Academic Research Consortium, BARC = Bleeding Academic Research Consortium CK-MB = Creatine Kinase-Myocardial Band , ECG = Electrocardiography, GUSTO = Global Utilization Of Streptokinase And Tpa For Occluded Arteries, ST = Stent Thrombosis, TIMI = Thrombosis in Myocardial Infarction, URL = Upper Reference Limit.

**References**

1. Baber U, Mehran R, Giustino G, Cohen DJ, Henry TD, Sartori S, Ariti C, Litherland C, Dangas G, Gibson CM, Krucoff MW, Moliterno DJ, Kirtane AJ, Stone GW, Colombo A, Chieffo A, Kini AS, Witzenbichler B, Weisz G, Steg PG and Pocock S. Coronary Thrombosis and Major Bleeding After PCI With Drug-Eluting Stents: Risk Scores From PARIS. *J Am Coll Cardiol*. 2016;67:2224-2234.

2. Natsuaki M, Morimoto T, Yamaji K, Watanabe H, Yoshikawa Y, Shiomi H, Nakagawa Y, Furukawa Y, Kadota K, Ando K, Akasaka T, Hanaoka KI, Kozuma K, Tanabe K, Morino Y, Muramatsu T, Kimura T, Credo-Kyoto Pci/Cabg Registry Cohort R and investigators Nt. Prediction of Thrombotic and Bleeding Events After Percutaneous Coronary Intervention: CREDO-Kyoto Thrombotic and Bleeding Risk Scores. *J Am Heart Assoc*. 2018;7.

3. Costa F, van Klaveren D, James S, Heg D, Raber L, Feres F, Pilgrim T, Hong MK, Kim HS, Colombo A, Steg PG, Zanchin T, Palmerini T, Wallentin L, Bhatt DL, Stone GW, Windecker S, Steyerberg EW, Valgimigli M and Investigators P-DS. Derivation and validation of the predicting bleeding complications in patients undergoing stent implantation and subsequent dual antiplatelet therapy (PRECISE-DAPT) score: a pooled analysis of individual-patient datasets from clinical trials. *Lancet*. 2017;389:1025-1034.

4. Rozemeijer R, Stein M, Voskuil M, van den Bor R, Frambach P, Pereira B, Koudstaal S, Leenders GE, Timmers L, Rittersma SZ, Kraaijeveld AO, Agostoni P, Roes KC, Doevendans PA, Stella PR and ReCre8 Study I. Randomized All-Comers Evaluation of a Permanent Polymer Zotarolimus-Eluting Stent Versus a Polymer-Free Amphilimus-Eluting Stent. *Circulation*. 2019;139:67-77.
