# Supplementary material for: Direct comparison of predictive performance of PRECISE-DAPT versus PARIS versus CREDO-Kyoto: a subanalysis of the ReCre8 trial
Source: Neth Heart J. 2020 Sep 21;29(4):201–14. doi: 10.1007/s12471-020-01486-y (PMC7991032; doi:10.1007/s12471-020-01486-y)
Supplement: Supplementary file 5 — Tab. 4 Net Reclassification tables of contemporary risk scores [file 12471_2020_1486_MOESM5_ESM.docx]

**Electronic Supplementary Material**

**Tab. 4** Net Reclassification tables of contemporary risk scores

A: **Reclassification table PARIS ischemic vs CREDO-Kyoto ischemic**

|  | Cases |  |  |  | Non-cases |  |  |
| --- | --- | --- | --- | --- | --- | --- | --- |
|  |  |  | **CREDO-Kyoto** |  |  | **CREDO-Kyoto** |  |
|  |  | Low | IM | High | Low | IM | High |
|  | **Low** | 39 (33%) | **10 (9%)** | **2 (2%)** | 604 (44%) | **116 (8%)** | **13 (1%)** |
| PARIS | **IM** | **23** (20%) | 9 (8%) | **2 (2%)** | **358** (26%) | 75 (5%) | **17 (1%)** |
|  | **High** | **10** (9%) | **12 (10%)** | 9 (8%) | **101** (44%) | **65 (4%)** | 27 (2%) |

B: **Reclassification table PARIS bleeding vs CREDO-Kyoto bleeding**

|  | Cases |  |  |  | Non-cases |  |  |
| --- | --- | --- | --- | --- | --- | --- | --- |
|  |  |  | **CREDO-Kyoto** |  |  | **CREDO-Kyoto** |  |
|  |  | Low | IM | High | Low | IM | High |
|  | **Low** | 9 (8%) | **5 (4%)** | **3 (3%)** | 515 (37%) | **162 (12%)** | **13 (1%)** |
| PARIS | **IM** | **4 (3%)** | 4 (3%) | **3 (3%)** | **424 (31%)** | 184 (13%) | **42 (3%)** |
|  | **High** | **1 (1%)** | **2 (2%)** | 3 (3%) | **57 (4%)** | **41 (3%)** | 20 (1%) |

C: **Reclassification table PARIS bleeding vs PRECISE-DAPT**

|  | Cases |  |  |  | Non-cases |  |  |
| --- | --- | --- | --- | --- | --- | --- | --- |
|  |  |  | **PRECISE-DAPT** |  |  | **PRECISE-DAPT** |  |
|  |  | Low | IM | High | Low | IM | High |
|  | **Low** | 12 (10%) | **3 (3%)** | **0 (0%)** | 582 (42%) | **55 (4%)** | **12 (1%)** |
| PARIS | **IM** | **7 (6%)** | 1 (1%) | **2 (2%)** | **339 (25%)** | 116 (8%) | **144 (10%)** |
|  | **High** | **0 (0%)** | **0 (0%)** | 6 (5%) | **7 (1%)** | **15 (1%)** | 96 (7%) |

D: **Reclassification table CREDO bleeding vs PRECISE-DAPT**

|  | Cases |  |  |  | Non-cases |  |  |
| --- | --- | --- | --- | --- | --- | --- | --- |
|  |  |  | **PRECISE-DAPT** |  |  | **PRECISE-DAPT** |  |
|  |  | Low | IM | High | Low | IM | High |
|  | **Low** | 8 (7%) | **3 (3%)** | **2 (2%)** | 674 (49%) | **121 (9%)** | **140 (10%)** |
| CREDO-Kyoto | **IM** | **6 (5%)** | 0 (0%) | **3 (3%)** | **224 (16%)** | 52 (4%) | **82 (6%)** |
|  | **High** | **5 (4%)** | **1 (1%)** | 3 **(3%)** | **30 (2%)** | **13 (1%)** | 30 (2%) |

Post-discharge event patients were considered cases, whereas patients without events were considered non-cases. Values on the diagonal (top left to bottom right) indicate that risk scores agreed on the classification of these patients. Values in bold indicate disagreement between the risk scores.
