# Supplementary material for: Direct comparison of predictive performance of PRECISE-DAPT versus PARIS versus CREDO-Kyoto: a subanalysis of the ReCre8 trial
Source: Neth Heart J. 2020 Sep 21;29(4):201–14. doi: 10.1007/s12471-020-01486-y (PMC7991032; doi:10.1007/s12471-020-01486-y)
Supplement: Supplementary file 6 — Tab. 5 Net benefit tables for contemporary risk scores [file 12471_2020_1486_MOESM6_ESM.docx]

**Electronic Supplementary Material**

**Tab. 5** Net benefit tables for contemporary risk scores

Net benefit for post-discharge ischemic events at different thresholds:

| Risk threshold | Net benefit using all as low risk | Net benefit using all as high risk | Net benefit of using PARIS | Net benefit of using CREDO-Kyoto |
| --- | --- | --- | --- | --- |
| 0% | 0% | 3.7% | 3.7 | 3.7 |
| 1% | 0% | 2.8% | 2.8% | 2.8% |
| 2% | 0% | 1.7% | 1.7% | 1.7% |
| 3% | 0% | 0.7% | 0.7% | 1.3% |
| 4% | 0% | -0.3% | 0.5% | 1.0% |
| 5% | 0% | -1.3% | 0.2% | 0.7% |
| 6% | 0% | -2.4% | 0.1% | 0.6% |
| 7% | 0% | -3.5% | 0.1% | 0.4% |
| 8% | 0% | -4.6% | 0.1% | 0.2% |
| 9% | 0% | -5.8% | 0.1% | 0.1% |
| 10% | 0% | -6.9% | 0.0% | 0.1% |

**Net benefit for post-discharge ischemic events** = (true-positive classifications – [% risk threshold/(100 − % risk threshold) × false-positive classifications])/total number of participants.

The number of additional true positives per 100 patients that the risk scores can identify without additional false positives is calculated as follows:

([% net benefit of using the score of interest] – [% net benefit of the alternative strategy])/([% risk threshold/100 − [risk threshold]).

This value is reflects the reduction in false positives without a decrease in the number of true positives.

Net benefit for post-discharge bleeding events at different thresholds is calculated as:

| Risk threshold | Net benefit using all as low risk | Net benefit using all as high risk | Net benefit of using PARIS | Net benefit of using PRECISE-DAPT | Net benefit of using CREDO-Kyoto |
| --- | --- | --- | --- | --- | --- |
| 0% | 0% | 2.1% | 2.1% | 2.1% | 2.1% |
| 1% | 0% | 1.2% | 1.2% | 1.2% | 1.2% |
| 2% | 0% | 0.2% | 0.4% | 0.1% | 0.3% |
| 3% | 0% | -0.9% | 0.2% | 0.1% | 0.5% |
| 4% | 0% | -1.9% | 0.0% | 0.0% | 0.4% |
| 5% | 0% | -3.0% | 0.0% | 0.0% | 0.3% |
| 6% | 0% | -4.1% | 0.0% | 0.0% | 0.3% |
| 7% | 0% | -5.2% | 0.0% | 0.0% | 0.2% |
| 8% | 0% | -6.4% | 0.0% | 0.0% | 0.2% |
| 9% | 0% | -7.5% | 0.0% | 0.0% | 0.1% |
| 10% | 0% | -8.7% | 0.0% | 0.0% | 0.1% |

**Net benefit for post-discharge bleeding events =** (true-positive classifications – [% risk threshold/(100 − % risk threshold) × false-positive classifications])/total number of participants.

The number of additional true positives per 100 patients that the risk scores can identify without additional false positives is calculated as

follows: ([% net benefit of using the score of interest] – [% net benefit of the alternative strategy in question])/([% risk threshold/100 − [risk threshold]).

This value is the equivalent to the reduction in false positives without a decrease in the number of true positives.
